# Characterizing local pig breeds as reservoirs for the domestic pig genetic variability worldwide via contributions to gene diversity and allelic richness

**DOI:** 10.1093/jas/skae329

**Published:** 2024-10-26

**Authors:** Katherine D Arias, Iván Fernández, Juan Pablo Gutiérrez, Riccardo Bozzi, Isabel Álvarez, Félix Goyache

**Affiliations:** Área de Genética y Reproducción Animal, SERIDA-Deva, Gijón, Asturias, Spain; Área de Genética y Reproducción Animal, SERIDA-Deva, Gijón, Asturias, Spain; Departamento de Producción Animal, Universidad Complutense de Madrid, Madrid, Spain; DAGRI, Università degli Studi di Firenze, Firenze, Italy; Área de Genética y Reproducción Animal, SERIDA-Deva, Gijón, Asturias, Spain; Área de Genética y Reproducción Animal, SERIDA-Deva, Gijón, Asturias, Spain

**Keywords:** domestic pig diversity, pig metapopulation worldwide, gene diversity, allelic richness, genetic reservoirs, ascertainment bias

## Abstract

Contributions to gene diversity and allelic richness were computed in a total of 2,260 domestic pig genotypes, sharing 53,626 autosomal SNPs, belonging to 98 pig subpopulations worldwide (41 Asian, 696 genotypes; 20 American, 262 genotypes; and 37 European, 686 genotypes), using 616 pig samples belonging to 5 different Cosmopolitan pig breeds as an outgroup, to ascertain if local pig subpopulation can be considered reservoirs of genetic diversity for the whole domestic pig species worldwide. Assessments were carried out for the whole dataset and separately for the American-European and Asian subsets. Effective population size was computed at the subpopulation level using molecular coancestry and linkage disequilibrium information to ensure that estimates of contributions to diversity were not affected by demographic issues. Most American and European pig subpopulations tended to have favorable contributions to both gene diversity and allelic richness. However, contributions to allelic richness were more consistent than those obtained for gene diversity, whether the computations are performed using either the whole dataset or the American–Asian subset, suggesting that allelic richness can be a key parameter to identify putative reservoirs for the species. The Asian pig subpopulations never contributed favorably to the allelic richness of the domestic pig metapopulation. Although these results can partially be explained by the highly divergent origins of the American-European and the Asian pig subpopulations, it cannot be discarded that the results obtained for the Asian subpopulations are biased due to a worse calling performance of the pig SNP arrays used for genotyping. The use of other potentially less biased sources of genotypic information is advisable to compare the Asian and American-European pig subpopulations genetic diversity.

## Teaser

Analyses showed large differences in contributions to gene diversity and allelic richness between Asian and American-European pig subpopulations. However, such differences may partially result from differences in performance of the SNP arrays when applied to either Asian or European-related pig samples.

## Introduction

Despite the historically extensive gene flow experienced among them, local populations of domestic pigs show large phenotypic variability and may act as reservoirs for the genetic diversity of the species (Megens et al., 2008). The domestic pig scenario currently includes feral populations, local pig breeds usually managed within breeding organizations, and highly selected cosmopolitan pig breeds and commercial lines. Geographical differences in domestic pig genetic diversity stem from a complex domestication process. Further than the assumption that European and Asian domestic pigs derive from 2 different domestication events (Yang et al., 2017), genetic evidence suggests that there existed multiple centers of domestication in which different human populations, mastering the domestication technology, recruited pig individuals from the wild either as a primary event or as a secondary event by substitution via crossbreeding of the pre-extant domesticated stock (Larson et al., 2005, 2007a, 2007b). Subsequent human-mediated dispersion and adaptation to different environments and different needs have contributed to shaping the domestic pig diversity. Recently, in historical terms, an intense genetic selection of local pig populations, mainly in the UK as well as Northern and Central Europe, has produced a set of improved pig breeds that have now spread worldwide and can now be considered cosmopolitan (Buchanan and Stalder, 2011). Many Southern and Midlands English pig breeds, some of which have become cosmopolitan, are believed to have been crossbred with Asian pigs to a large extent (Megens et al., 2008). Cosmopolitan pig breeds did not influence China until recently. However, in the last 2 decades of the 20th century, Cosmopolitan pig breeds were introduced into China putting local Chinese pig genetic resources at risk (Yang et al., 2003). Moreover, it is not always clear whether, and to what degree, historical or recent interactions between breeds have affected their uniqueness. This applies especially to poorly documented local populations, which gradually merge into neighboring populations.

Genetic diversity in a population can be influenced by several demographic factors, such as selection, isolation, or between-populations migration (Hague and Routman, 2016). Although most of these factors can be summarized into effective population size that can be inferred from molecular information (Santiago et al., 2024), the computation of contributions to diversity for each subpopulation (e.g., breed) in a metapopulation allows a direct between-populations comparison. Contributions to diversity were formerly assessed for limited sets of pig subpopulations using the phylogenetic-like Weitzman (1992) approach (Ollivier et al., 2005; Cortés et al., 2016). However, this recursive method makes unwarranted assumptions (Faith, 1994). Furthermore, despite attempts to fill this gap (Ollivier and Foulley, 2005), the Weitzman (1992) approach does not explicitly use within-population diversity. Methods considering gene diversity (expected heterozygosity; Caballero and Toro, 2002) and allelic richness adjusted (rarefacted) for sample size (Petit et al., 1998) decompose both parameters into within- and between-population fractions within a metapopulation and have the advantage of straightforward interpretation: gene diversity illustrates the existence of balanced allelic frequencies in a subpopulation and allelic richness can characterize the degree of genetic uniqueness or distinctiveness of a subpopulation (Petit et al., 1998; Caballero and Toro, 2002). Both parameters are important for small or endangered populations. Although they provide highly relevant information on their evolutionary potential, long-term response to selection, the occurrence of bottlenecks, and changes in population size (see López-Cortegano et al., 2019 for a review), these 2 parameters give rather different insights, allowing conservation strategies to benefit from complementary diversity measures (Caballero and García-Dorado, 2013).

In the global scenario of pig breeding, this research aims at answering the following question: can local pig breeds in Europe, America, and Asia be considered reservoirs of genetic diversity for the whole pig population worldwide? To deal with this task, marginal contributions to gene diversity (Caballero and Toro, 2002) and allelic richness (Petit et al., 1998) will be computed in a total of 98 local pig populations (gathering 1,644 genotypes) using as reference 616 genotypes of Cosmopolitan pig genotypes belonging to 5 different breeds.

## Materials and Methods

### Ethical statement

Most genotypes used in this research were obtained from public repositories according to the corresponding national regulations of animal care and ethics in research (Ai et al., 2013; Burgos-Paz et al., 2013; Goedbloed et al., 2013; Iacolina et al., 2016; Yang et al., 2017; Scandura et al., 2022). New genotypes were obtained in the laboratories of SERIDA (Arias et al., 2024). SERIDA adheres to the Ethical Committee in Research of the University of Oviedo (Spain), which ensures that all research with biological agents follows Good Laboratory Practices and European and Spanish regulations on biosecurity under the Regulation of February 13, 2014 (BOPA no. 47 on February 26, 2014). Blood and hair root samples used in this project were collected by veterinary practitioners following standard procedures and relevant national guidelines to ensure appropriate animal care, with the permission and in the presence of the owners. For this reason, permission from the Ethical Committee in Research of the University of Oviedo was not required.

### Available datasets

A total of 2,299 domestic pig genotypes were obtained using the PorcineSNP60 BeadChip genotyping platform (Illumina Inc., San Diego, CA, USA) belonging to 102 different pig subpopulations, including local Asian, local American, local European, and cosmopolitan pig subpopulations, were retrieved from public repositories (Ai et al., 2013; Burgos-Paz et al., 2013; Goedbloed et al., 2013; Iacolina et al., 2016; Yang et al., 2017; Scandura et al., 2022). Data were edited to remove those local pig populations with sample sizes lower than 6 individuals. Finally, the dataset retrieved from public repositories included: 36 local European pig subpopulations sampled in a total of 13 different countries; local American pig genotypes belonging to 4 local American pig subpopulations (83 individuals sampled in different locations of the USA), 13 American Creole (143 genotypes); local American pig subpopulations of assumed origin in the Iberian Peninsula; (Burgos-Paz et al., 2013; Cortés et al., 2016), and 3 American feral pig samples (36 genotypes); local Asian pig genotypes belonging to 37 Chinese pig subpopulations, one Korean local, and 3 local Thai pig subpopulations. Regarding Cosmopolitan pigs, up to 578 domestic pig genotypes belonging to 6 different subpopulations (Duroc, Hampshire, Landrace, Large White, Pietrain, and Berkshire) were sampled in the USA (158), Canada (20), and 9 different European countries (400) were obtained and pooled to be used as reference.

Furthermore, 167 genotypes previously analyzed in Arias et al. (2024), were filtered for the presence of null and partially null alleles due to technical issues by fitting *F*_*IS*_ > 0.9 as a threshold (Arias et al., 2022) for populations in which pedigrees are not available, including 19 new Gochu Asturcelta and 6 Porco Celta Galego pig samples genotyped using the Axiom-PorcineHDv1 array (Affymetrix, Inc. Santa Clara, CA, USA; 658,692 SNPs) were available. This set included Spanish Gochu Asturcelta (41), Portuguese Bísara (19), Porco Celta Galego (6), Spanish Iberian (10), Portuguese Alentejano (7), Hampshire (5), Landrace (13), Large White (20), Italian Cinta Senese (26) and Korean local (20) pig genotypes. Genotypes of different origins belonging to the same pig breed were merged into a single subpopulation. Following previous approaches (Menéndez et al., 2016c; Arias et al. 2024), Spanish Iberian and Portuguese Alentejano samples were merged into one only “Iberian pig” subpopulation.

The full list (and their descriptors) of the samples used in analyses is given in Supplementary Table 1. Altogether, the analyzed data set comprised a total of 2,260 genotypes belonging to 98 local pig subpopulations (41 Asian, 696 genotypes; 20 American, 262 genotypes; and 37 European, 686 genotypes) and an outgroup formed by 616 Cosmopolitan pig individuals.

### Imputation process

Although all genotypes downloaded from public repositories were obtained using the Illumina PorcineSNP60 BeadChip, the number of available SNPs varied across projects from 44,462 (Ai et al., 2013) to 51,254 (Yang et al., 2017). A total of 34,300 autosomal SNPs were shared across projects and further used as a target for imputation using BEAGLE (Browning et al., 2018, 2021). The Axiom-PorcineHDv1 genotypes were used as reference sets for imputation. The dataset provided by Ai et al. (2013) was previously subject to quality control measures (call rate ≥ 90% and MAF ≥ 0.05) and was used as a template for the other publicly available datasets to avoid applying recurrent filters.

Imputation was carried out as follows: first, missing genotypes of both the reference and the target sets were imputed with default parameters; second, both the reference and the target sets were split into 2 reference and target subsets (Asian and non-Asian) to improve the imputation process via decreasing the genetic differentiation between the reference and the target populations (Pryce et al., 2014; Ding et al., 2023); finally, imputation was carried out separately for either the Asian and the non-Asian subsets. Following Liu et al. (2012), only SNPs with DR^2^ ≥ 0.9 (estimated squared correlation between each imputed genotype and its true underlying genotype) were retained. Inconsistencies in strand orientation between platforms were resolved using the *--flip* function of PLINK v1.9 (Chang et al., 2015). Finally, all individuals used for further analyses had 53,626 autosomal SNPs.

### Population structure analyses

The program PLINK v1.9 (Chang et al., 2015) was used to compute principal component analysis (PCA). Eigenvectors computed for each individual were averaged by subpopulation and used to construct dispersion plots using the library ggplot2 of R (http://CRAN.R-project.org/).

A cluster analysis was carried out on the genotypes of the pig metapopulation studied using the program Admixture v1.23 (Alexander et al., 2009; Alexander and Lange, 2011), which calculates maximum-likelihood estimates of individual ancestries based on data provided by multiple loci. Analyses were conducted for 2 ≤ *K* ≤ 20, being *K* the number of clusters given the data. The optimal number of clusters was determined by performing the cross-validation procedure included in the program Admixture. By default, the procedure partitions all the observed genotypes into 5 roughly equally sized folds for each *K*. Within *K*, each fold was used as a test set, while the other 4 were used for training. For each fold, prediction error is estimated by averaging the squares of the deviance residuals for the binomial model. Admixture barplots were constructed using the program CLUMPAK (Kopelman et al., 2015).

### Contributions to diversity

All cosmopolitan pig genotypes were merged into one population only and used as an outgroup for diversity analyses. The subpopulations were the target for computations. Subpopulations’ contributions to diversity were assessed using a) the whole dataset; and b) separately for the Asian and American-European pig subpopulations. When necessary for descriptive purposes, local pig populations within the continent were merged by geographical area or expected origin into the following groups: American Creole, American Feral (feral or semi-feral pig subpopulations), Other American (the remaining American pig subpopulations), Chinese subpopulations, Other Asian (Thai and Korean local pig subpopulations), Mediterranean (local Portuguese, Spanish, and Italian pig subpopulations), United Kingdom (UK) subpopulations, and Other European (Central, Northern and East Europe pig subpopulations).

Contributions to gene diversity (Nei, 1987) and rarefacted allelic richness (Hurlbert, 1971) were assessed using the program Metapop2 (López-Cortegano et al., 2019) following the methods by Caballero and Toro (2002) and Petit et al. (1998), respectively. Both approaches explicitly use within-population and between-subpopulations variation to identify those subpopulations contributing the most to the overall genetic diversity of a metapopulation. The contribution of each subpopulation was estimated by sequentially removing them and re-calculating the changes in within-subpopulation diversity, between-subpopulations diversity, and total diversity (López-Cortegano et al., 2019). Both within (HS) and between (Nei’s minimum genetic distance, DG) subpopulations gene diversity were computed to define the total gene diversity as HT = HS + DG. Analogously, total allelic diversity (AT) was computed using within-subpopulation (AS) and between-subpopulations (DA) allelic richness estimates as AT = AS + DA. The AS component was computed from the average number of alleles segregating in the subpopulations minus one. The DA component was calculated based on the average number of unique alleles present in the subpopulation compared with other subpopulations. A description of the formulas used for computations can be found elsewhere (Bozzi et al., 2012; Jordana et al., 2017). The implementation of Caballero and Toro’s (2002) method in the program Metapop2 (López-Cortegano et al., 2019) allows an intuitive interpretation of its results is consistent with those obtained for Petit et al.’s (1998) method: positive contributions for both gene diversity and allelic richness mean that the subpopulation assessed contributes favorably to the metapopulation’s diversity.

### Computation of effective population size

For each subpopulation in the dataset, estimates of contemporary effective size (*N*_*e*_) were obtained from molecular information using the Linkage Disequilibrium (*N*_*e*(LD)_) and the molecular coancestry (*N*_*e*(M)_) approaches implemented in the program *NeEstimator* v2.1 (Do et al., 2014). *NeEstimator* uses a jackknife procedure to construct 95% confidence intervals of the estimates. Estimates of *N*_*e*(LD)_ were obtained using the Waples’ (2006) approach, which corrects for biases resulting from the presence of rare alleles, assuming random mating and removing alleles with frequencies (*Pcrit*) lower than 0.05. Estimates of *N*_*e*(M)_ were obtained using the method proposed by Nomura (2008), which uses alleles at any frequency for computations and applies the correction suggested by Oliehoek et al. (2006) for increasing the importance of loci with small expected homozygosity and balanced allele frequencies.

To decrease computation effort, dataset was pruned using the *--indep-pairwise* option of the PLINK v1.9 software with the following parameters (Yang et al., 2024): 50 SNPs per window, a shift of 10 SNPs between windows at each step, and a pairwise *r*^*2*^ threshold of 0.2. A total of 9,410 SNPs were retained and further used for *N*_*e*_ estimations.

### Statistical relationships between estimates

The local pig populations were analyzed in the whole data set and the 2 geographical subsets (either American-European and Asian pig subpopulations) were classified into 2 or 3 classes according to sample size a) local populations with sample size < 10 and local populations with sample size ≥ 10; b) local populations with sample size < 20 and local populations with sample size ≥ 20; and c) local populations with sample size < 10, local populations with sample size > 9 and lower than 20, and local populations with sample size ≥ 20. Further, the analyzed pig populations were classified according to the sign (positive or negative) of the HT and AT contributions to diversity following the sign (positive or negative) of the HT and AT contributions to diversity: a) positive (favorable) contributions to both HT and AT; b) positive contributions to HT and negative contributions to AT; c) negative (unfavorable) contributions to both HT and AT; and d) negative contributions to HT and positive contributions to AT. The classes fitted for sample size and sign of contributions to diversity were statistically contrasted using the Chi-square Mantel–Haenszel test, as implemented in the Proc Freq of SAS/STAT (SAS Inst. Inc., Cary, NC).

Furthermore, Spearman rank and Pearson correlations between *N*_*e*(LD)_ and *N*_*e*(M)_ estimates, between the within-population (HS and AS), between-populations (DG and DA), and total contributions (HT and AT) to gene diversity and allelic richness and between estimates of *N*_*e*_ and contributions to diversity were computed using the Proc Corr of SAS/STAT (SAS Inst. Inc., Cary, NC).

## Results

The contributions to diversity, effective size, and mean PCA eigenvalues computed using the analyzed dataset, as well as a geographical description of the subpopulations, are given by subpopulation in Supplementary Table 2.

### Population structure

**Figure. 1.**
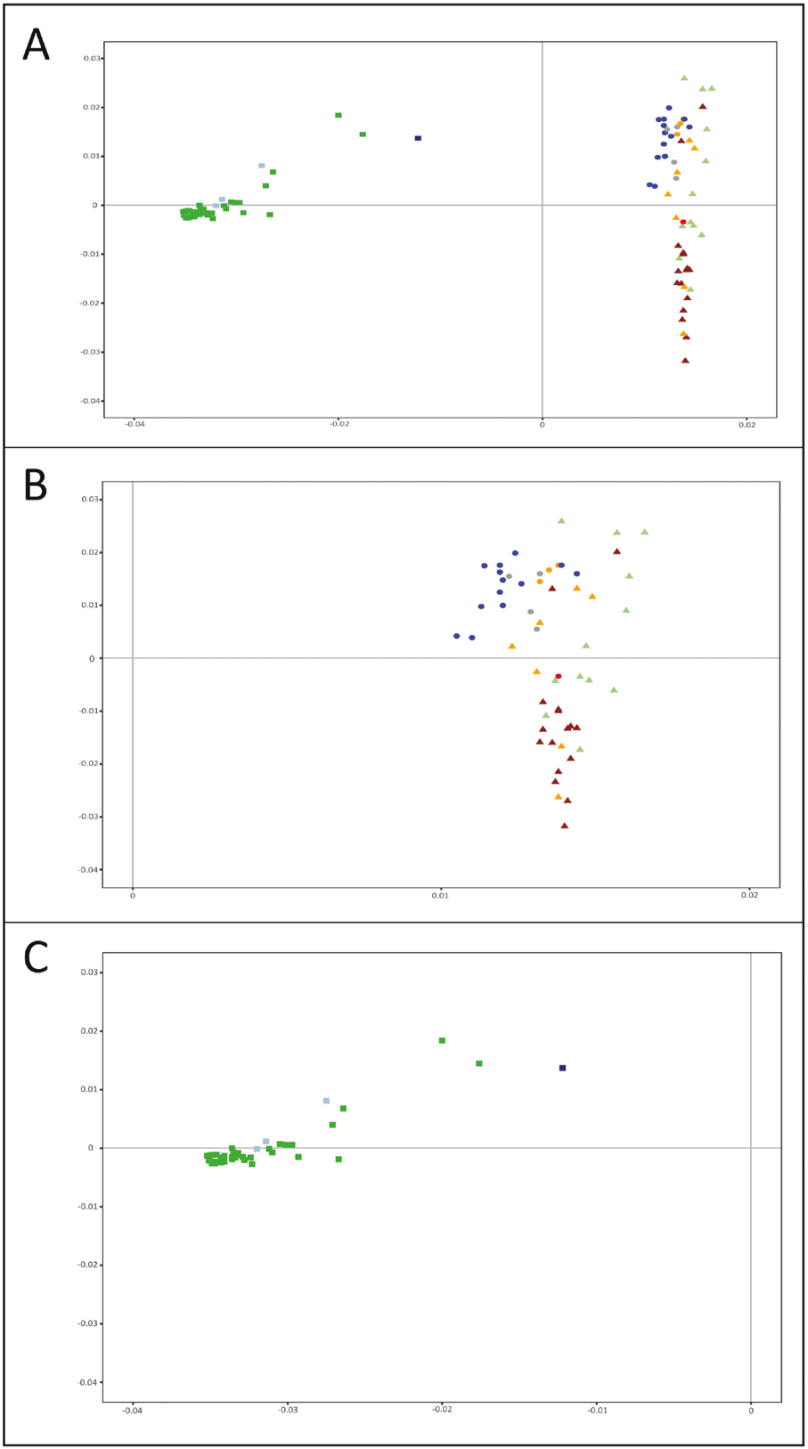
Dispersion plots summarizing the between-subpopulation relationships assessed via PCA. Factor 1, explaining 56% of the total variability is on the X-axis. Factor 2, explaining 9% of the total variability is in the Y-axis. The Cosmopolitan pig outgroup is in a red circle. American Creole pig subpopulations are in blue circles, American Feral subpopulations in orange circles, and Other American pig subpopulations in gray circles; Chinese subpopulations are in green squares, Thai pig subpopulations in light blue squares, and the Korean Local subpopulation in dark blue square; Mediterranean pig subpopulations are in light green triangles, United Kingdom (UK) pig subpopulations in orange triangles, and Other European pig subpopulations are in brown triangles. Plot A illustrates the relationships assessed for the whole dataset whereas Plots B and C zoom up the American-European and the Asian scenarios, respectively.

Figure 1 illustrates the between-subpopulations relationships in the analyzed dataset assessed via PCA. On the X-axis, PC1 (explaining 56% of the total variability) separates Asian pig subpopulations from the European and American pig subpopulations (Fig. 1A). On the Y-axis, PC2 (9% of the total variability) separates the American and a part (12) of the European pig subpopulations including the Iberian-related Mediterranean pig subpopulations. Moreover, on the positive values of the Y-axis, a few Central European subpopulations, such as the Hungarian Mangalitsa, were separated from most (23) Central, Northern, and Eastern European pig subpopulations, the Cosmopolitan outgroup, and some Mediterranean pig subpopulations including those assigned to the Celtic-Iberian pig strain. A more detailed survey of the non-Asian pig subpopulations (Fig. 1B) informs on the fact that American Creole, American Feral, Iberian pig strain subpopulations (Iberian and Manchado de Jabugo), and most Italian pig subpopulations clustered on the positive values of the Y-axis. In contrast, most Central and Northern European subpopulations cluster with subpopulations belonging to the Celtic-Iberian pig strain (Portuguese Bísara, Spanish Gochu Asturcelta, and Porco Celta Galego) in the negative values of the Y-axis. UK pig subpopulations were highly dispersed among groups. Asian pig subpopulations (Fig. 1C) tended to cluster together on the X-axis with no clear separation between Thai and Chinese pig subpopulations. The only exception was observed for the Korean Local and 2 Chinese pig subpopulations (the composite Sutai and the northern China Lichahei subpopulations).

**Figure. 2.**
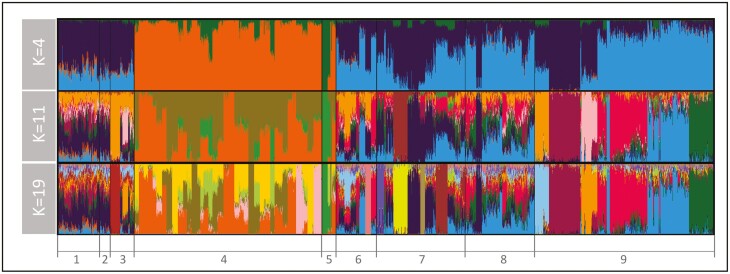
Barplots summarizing admixture analyses carried out using 1,644 local pig genotypes belonging to 98 different subpopulations and 616 Cosmopolitan pig samples. The results are plotted according to the 8 different pig groups (1: Creole; 2: Feral; 3: Other American; 4: China; 5: Other Asian; 6: UK; 7: Mediterranean; 8: Other European) fitted within Continent and the Cosmopolitan outgroup (9). The individual ancestries estimated using Admixture v1.23 were illustrated for *K* = 4, *K* = 11, and *K* = 19.

Admixture results were added to those obtained with PCA (Fig. 2). The lowest cross-validation error was at *K* = 19 (Supplementary Figure 1). However, the increase in error between *K* = 11 and *K* = 12 was negligible (lower than 0.001). Therefore, barplots corresponding to *K* = 11 and *K* = 19 are shown in Figure 2 together with that of *K* = 4 to allow a general overview of the partition of the genetic backgrounds represented in the metapopulation under study.

On *K* = 4, it can be assessed that the Asian pig subpopulations are separated from the others (American, European, and Cosmopolitan), which, in turn, tend to share 2 main different genetic backgrounds: Cosmopolitan, in light blue, and non-Cosmopolitan, in dark blue. On *K* = 11, the Cosmopolitan admixed population used as an outgroup split into different clusters (one per Cosmopolitan breed used). On *K* = 19, it is worth mentioning that Mediterranean pig subpopulations split into very different clusters that do not always relate with geography: some southern (Calabrese pig breed in light purple), central (Cinta Senese pig breed in yellow), and northern (Mora Romagnola pig breed in khaki color) Italian Mediterranean pig subpopulations can be differentiated among them and from the Spanish Iberian genetic background (dark blue). Moreover, the Hungarian Mangalitsa pig breed and, partially, the American Creole pig subpopulations shared genetic background with the Iberian pig strain.

### Contributions to diversity

For descriptive purposes, diversity parameters and contributions to diversity computed at the pig group level within continents are given in Table 1. The Cosmopolitan pig pool had the higher gene diversity (0.365). Among groups, this parameter varied from 0.192 (Chinese pigs) to 0.377 (Other European pigs). The 2 Asian groups had the higher values for gene diversity and the lower for allelic richness (1.7). The more favorable contribution to gene diversity was assessed for the Cosmopolitan pig outgroup (4.457). Among groups, the less favorable total contribution to gene diversity was assessed for the 4 non-Chinese pigs (−0.189) whereas the more favorable (2.136) was assessed for the Chinese group due to a very high between-subpopulations differentiation component (15.073). Regarding allelic richness, the more favorable total contribution was assessed for the Mediterranean pig group (0.603), having the higher within-subpopulations component (1.024), whereas the less favorable were assessed for the Asian pig groups (−0. 892 and −0.800), caused by very low within-subpopulations components (−3.366 and −2.592).

**Table 1.** Within−subpopulation, between−subpopulations, and total contributions to diversity computed using gene diversity (HS, DG, and HT, respectively) and allelic richness (AS, DA, and AT, respectively) for each pig group within continent. Additionally, the number of subpopulations per group and mean values for gene diversity and allelic richness within group are given

Continent	Group	*N*	Gene diversity	Allelic richness	Contributions to gene diversity and allelic richness					
					HS	DG	HT	AS	DA	AT
America	Creole	13	0.365	2.0	0.862	−0.569	0.293	0.885	−0.556	0.328
	Feral and semi-feral	3	0.340	2.0	0.100	−0.090	0.010	0.821	−0.522	0.299
	Other Americans^a^	4	0.326	2.0	0.099	−0.033	0.066	0.451	−0.256	0.195
Asia	China	37	0.192	1.7	−12.938	15.073	2.136	−3.366	2.475	−0.892
	Other Asians^b^	4	0.241	1.7	−0.409	0.220	−0.189	−2.592	1.792	−0.800
Europe	United Kingdom	7	0.360	2.0	0.767	−0.502	0.264	0.792	−0.499	0.293
	Mediterranean^c^	12	0.340	2.0	0.971	−0.659	0.312	1.024	−0.422	0.603
	Other Europeans^d^	17	0.377	2.0	1.900	−1.074	0.826	0.900	−0.562	0.338
Cosmopolitan			0.365	2.0	7.466	−3.009	4.457	1.086	−0.535	0.551
Total			0.323	1.9						

^a^Non−Creole and non−feral pig populations.

^b^Thai and Korean local pig populations.

^c^Local pig breeds native of the Iberian Peninsula and Italy.

^d^Central, Northern and Eastern Europe local pig breeds.

**Figure. 3.**
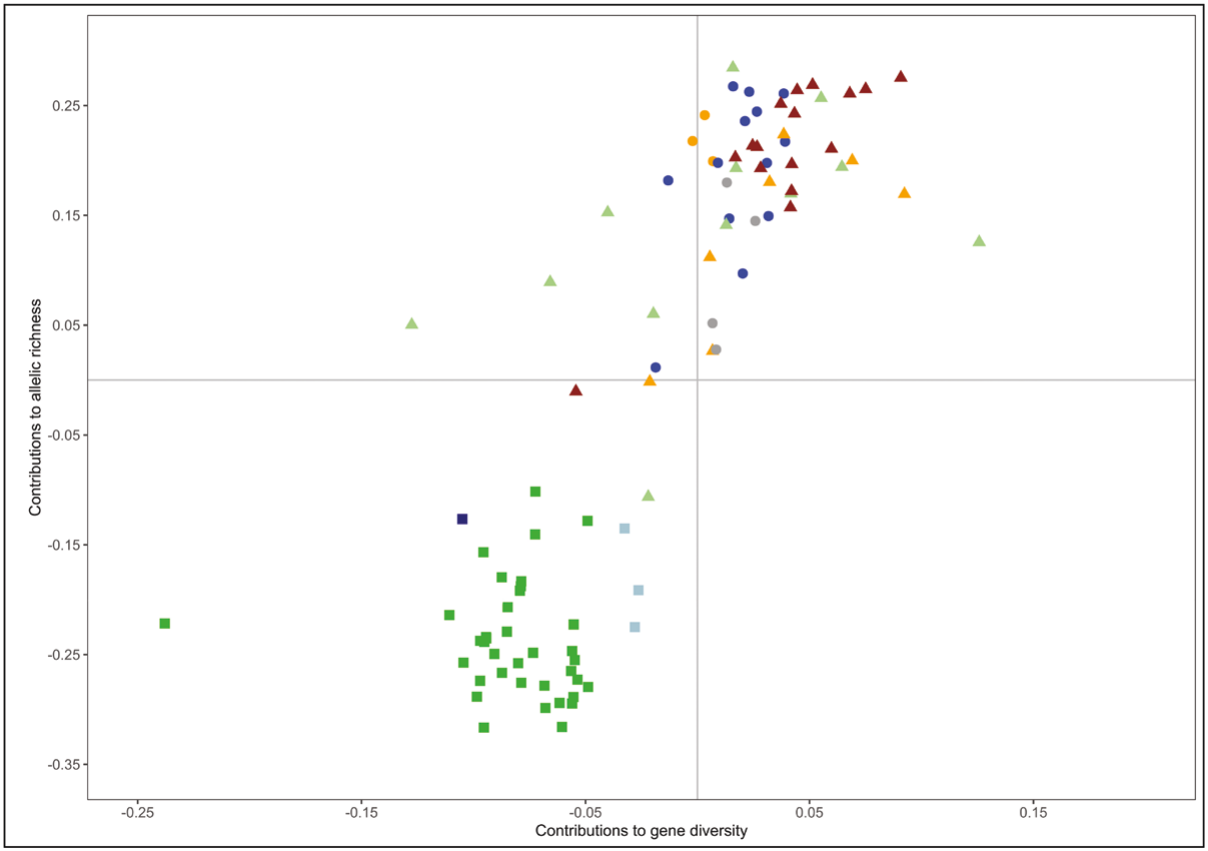
Dispersion plot illustrating the subpopulations contributions to gene diversity (X-axis) and allelic richness (Y-axis) assessed using the whole dataset. The Cosmopolitan pig outgroup was not plotted. American Creole pig subpopulations are in blue circles, American Feral subpopulations in orange circles and Other American pig subpopulations in gray circles; Chinese subpopulation are in green squares, Thai pig subpopulations in light blue squares and the Korean Local subpopulation is in dark blue square; Mediterranean pig subpopulations are in light green triangles, UK pig subpopulations in orange triangles, and Other European pig subpopulations are in brown triangles.

Values of marginal contributions to diversity assessed at the local pig subpopulation level were low. These results are detailed in Supplementary Table 2, and the relationships between the contributions to gene diversity and allelic richness in the local pig subpopulations analyzed are summarized in Figure 3. Excluding the Cosmopolitan pig outgroup and from more to less favorable, HT ranged from 0.126 (Gochu Asturcelta pig) to −0.238 (Tibetan pig), and AT ranged from 0.285 (Sardinian semiferal-pig) to −0.317 (Chinese Jinhua pig). The 41 Asian pig subpopulations analyzed had negative (unfavorable) contributions to both gene diversity and allelic richness. All Asian local pig subpopulations (on the bottom left quadrant of Figure 3) had unfavorable contributions to both gene diversity and allelic richness. The upper right quadrant of Figure 3, including most European and American pig subpopulations, gathers those pig subpopulations contributing favorably to both gene diversity and allelic richness. All American pig subpopulations had favorable (positive) total contributions to allelic richness. On the upper left quadrant of Figure 3, 3 American pig subpopulations (Peruvian Creole, Argentinian Semi-feral, and Yucatan minipig) and 4 European local pig subpopulations (Spanish Manchado de Jabugo, Iberian pig, Italian Cinta Senese, and Nera Siciliana) had unfavorable contributions to gene diversity. Only 3 European pig subpopulations (Mora Romagnola, Hungarian Mangalitsa, and UK Tamworth) had unfavorable (negative) marginal contributions to both gene diversity and allelic richness. It is worth noting that no local pig subpopulation contributed favorably to gene diversity and unfavorably to allelic richness (bottom right quadrant).

To account for possible differences due to population structure, contributions to diversity were re-calculated for both the European-American (Supplementary Table 3) and the Asian (Supplementary Table 4) subpopulations separately, always using the Cosmopolitan samples as outgroup. The range in which HT and AT varied within subsets: in the American-European subset, HT varied from −0.412 (Iberian) to 0.082 (Middle White), whereas AT varied from −0.293 (Tamworth) to 0.140 (Sardinian Feral); within the Asian subset HT varied from −1.090 (Tibetan) to −0.121 (Chinese Jhom Thong pig) whereas AT varied from −0.627 (Chinese Jinhua pig) to 0.967 (Korean local pig). Mainly due to their contributions to gene diversity, splitting the whole dataset into these 2 subsets caused a reorganization of the between-subpopulations relationships as well. Spearman correlation between the estimates of HT and AT obtained using the whole dataset and the continental subsets were 0.845 (*P* < 0.0001) and 0.992 (*P* < 0.0001) for the European-American pig subpopulations and 0.773 (*P* < 0.0001) and 0.990 (*P* < 0.0001) for the Asian pig subpopulations, respectively.

**Figure. 4.**
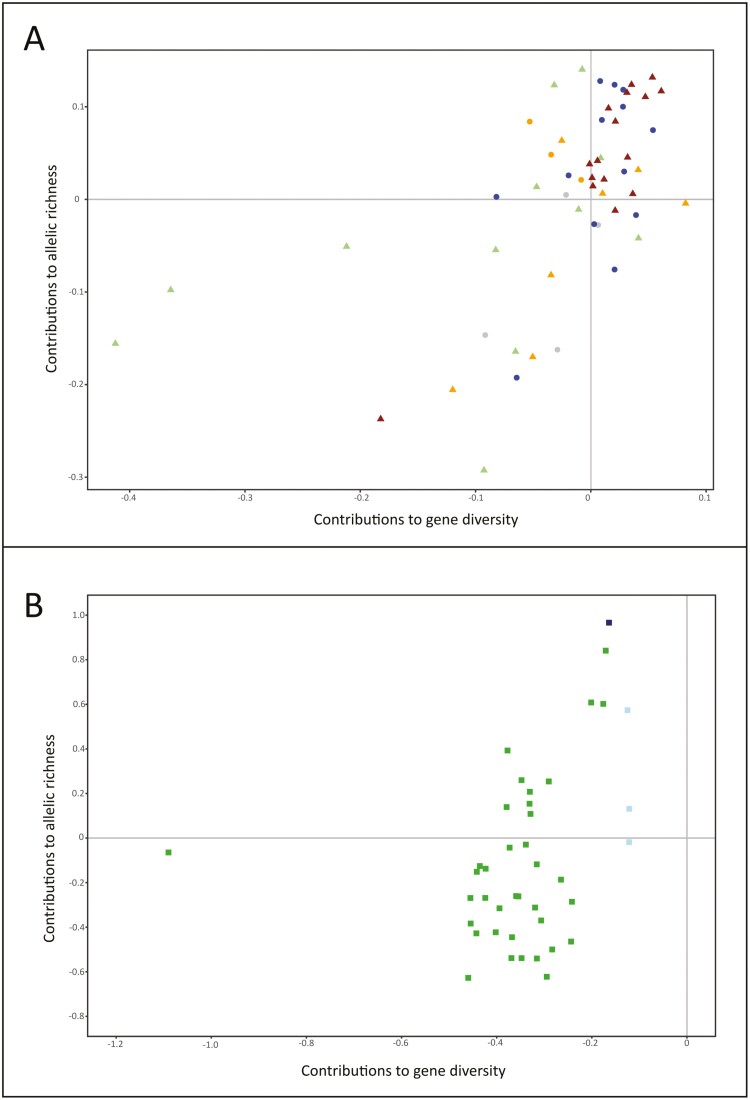
Dispersion plots illustrating the subpopulations contributions to gene diversity (X-axis) and allelic richness (Y-axis) assessed using the American-European (Plot A) and the Asian (Plot B) subsets. The Cosmopolitan pig outgroup was not plotted. American Creole pig subpopulations are in blue circles, American Feral subpopulations in orange circles and Other American pig subpopulations in gray circles; Chinese subpopulation are in green squares, Thai pig subpopulations in light blue squares and the Korean Local subpopulation is in dark blue square; Mediterranean pig subpopulations are in light green triangles, UK pig subpopulations in orange triangles, and Other European pig subpopulations are in brown triangles.

Figure 4A summarizes the results obtained for HT and AT using the European-American pig subpopulations subset. Most American Creole and Central, Northern, and Eastern European pig subpopulations contributed favorably to the whole American-European pig diversity (upper right quadrant). A second group of pig subpopulations formed by the American feral, 5 Mediterranean (including Iberian, Portuguese Bísara, Italian Sardinian semi-feral, Casertana, and Calabrese), and British Lop pig subpopulations contributed favorably to the allelic richness of the subset only (upper right quadrant of Figure 4A). Most pig subpopulations that contributed favorably to allelic richness only when the whole dataset was analyzed (Spanish Manchado de Jabugo, Italian Cinta Senese and Nera Siciliana, and Yucatán minipig), joined the pig subpopulations (Italian Mora Romagnola, Hungarian Mangalitsa, and UK Tamworth) previously assessed as unfavorable contributors to both gene diversity and allelic richness (bottom left quadrant). It is worth noting that several pig subpopulations, including the endangered Spanish Gochu Asturcelta and Swedish Linderöd, the endemic Ossabau, and the Brazilian Piau, contributed favorably to gene diversity but unfavorably to allelic richness.

Figure 4B summarizes the results obtained for HT and AT using the Asian pig subpopulations subset. All Asian pig subpopulations still had negative (unfavorable) contributions to gene diversity. However, besides the Thai and Korean local subpopulations, several Chinese pig subpopulations, including Northern China (such as Lichahei and Minzhu), Central-Southern China (Guandong, Leanhua, or Kele) or Tibetan (Milinzang) pig subpopulations, contributed favorably to allelic richness.

### Estimates of effective population size

Estimates of effective population size were computed for each subpopulation in the dataset (Supplementary Table 2). Values of *N*_*e(M)*_, ranging from 0.9 (Cinta Senese) to 10.4 (Argentinian Creole), were substantially lower than those obtained using linkage disequilibrium, ranging from 1.2 (Korean Local) to 373.3 (Argentinian Creole). Values of *N*_*e(LD)*_ showed larger 95% confidence intervals as well, with 4 subpopulations (Chinese JhomThong of Chiang Mai, Argentinian Semi-Feral, Russian Urzhum, and Argentinian Creole) including infinite in the upper limit of the corresponding 95% confidence intervals of *N*_*e(LD)*_. The narrower bounds of the 95% confidence intervals of *N*_*e(M)*_ suggested that these estimates of *N*_*e*_ may be more reliable.

### Correlation between estimates

Supplementary Table 5 summarizes the Spearman rank (*r*_*s*_) and Pearson (*r*) correlation between pairs of estimates of *N*_*e*_ and contributions to diversity. Correlations between estimates of *N*_*e*(M)_ and *N*_*e*(LD)_, even though statistically significant for *P* < 0.05, were low (*r*_*s*_ = 0.448; *r* = 0.546) for the whole dataset and even non-significant for the Asian pig subpopulations subset (*r*_*s*_ = 0.228; *r* = 0.180).

The total contributions to diversity (HT and AT) had higher coefficients for Spearman than for Pearson correlation, particularly for the whole dataset (*r*_*s*_ = 0.812; *r* = 0.259). The within-subpopulations (HS and AS) and between-subpopulations (DG and DA) components tended to follow the same pattern. However, Spearman and Pearson correlation coefficients tended to be more similar when the 2 subpopulation subsets were considered, with the Spearman correlation being very low for the pair DG–DA in the Asian subset.

Both the Spearman and the Pearson correlations computed between the estimates of *N*_*e*_ and the estimates of partial and total contributions to gene diversity and allelic richness were low, with *r*_*s*_ ranging from −0.381 (pair *N*_*e(M)*_—HS) to 0.379 (pair *N*_*e*(M)_–DG) and with *r* ranging from −0.369 (pair *N*_*e*(M)_–DG) to 0.379 (pair *N*_*e*(M)_–AS).

### Chi-square Mantel–Haenszel test

The Chi-square Mantel–Haenszel test informed that the classes constructed according to sample size and those constructed using the sign of the HT and AT contributions to diversity were not statistically associated. This was sequentially assessed in the whole dataset and in the 2 geographical subsets used whether the 2 or 3 sample size classes were fitted. In all cases, *P* was higher than 0.05, varying from *P* = 0.1286 for the American-European subset and 3 sample size classes to *P* = 0.9782 for the Asian subset and 2 sample size classes (<10 and ≥10).

## Discussion

The current research has been performed mirroring the present scenario of local domestic pigs worldwide, in which, whatever the continent and the country considered (Yang et al., 2003; Chen et al., 2017): a) Cosmopolitan pigs are present, and possibly influencing, in national pig stocks; b) a non-negligible proportion of European pig subpopulations may have been introgressed, directly or indirectly, with Asian pig genes in very different degrees; and c) extensive gene flow between ancestral local breeds has obscured the origin of modern breeds and the relationships between them. The use of commercial, here Cosmopolitan, pig genotypes to correctly quantify the genetic diversity of local pig populations has been suggested before (Faria et al., 2019).

Contributions to diversity of local pig subpopulations have been computed for the whole dataset but also splitting them into groups and continental subsets. However, it is worth noting that the results obtained using datasets of different compositions cannot be compared straightforwardly due to technical reasons: the Caballero and Toro’s (2002) method to assess contributions to gene diversity relies on the concept of metapopulations whereas the Petit et al.’s (1998) method to assess contributions to allelic richness is based on the concept of subpopulation. A substantial change of the allelic frequencies at the metapopulation level (e.g. removing Asian pig subpopulations from the dataset) affects both the contribution values assessed and the relative importance (rank position) of the subpopulation in the dataset. More importantly, in the case of allelic richness, merging various subpopulations into a single subpopulation is only acceptable as an academic approach useful for descriptive purposes. In our case, the splitting of the dataset into continental subpopulations kept the composition of the subpopulations the same, and a heavy reference (the 616 Cosmopolitan pig genotypes) was still used. This is why the subpopulations’ ranking order for parameter HT was not affected by the splitting into continental subsets.

Although the sample sizes of the analyzed subpopulations are not balanced, we consider that this fact does not significantly affect the scenario depicted by the computed contributions to diversity. Although losses in variability due to population bottlenecks or background selection, affecting allelic variant frequencies, could cause a reduction in *N*_*e*_ only (Charlesworth, 2009), *N*_*e*_ is useful for describing expected levels of genetic diversity, as a summary of the effect of different of demographic factors such as actual population census, shorter generation times, or differential rates of gene flow (Hague and Routman, 2016). Estimates of *N*_*e*_ computed using molecular coancestry (Nomura, 2008) and linkage disequilibrium (Waples, 2006) have a substantial divergence due to theoretical assumptions and methodological issues, namely the use of allelic variants in low frequency, with the *N*_*e*(M)_ estimates usually being lower than those obtained for the *N*_*e*(LD)_ (Menéndez et al., 2016b). In any case, Spearman rank correlations between either *N*_*e*(M)_ or *N*_*e*(LD)_ with estimates of contributions to the gene diversity and the allelic richness estimated using the whole dataset of the 2 geographical subsets fitted were low or very low, suggesting that these estimates are not dependent on effective population size. Further evidence suggesting that HT and AT are not dependent on actual sample size was obtained using Chi-square Mantel–Haenszel test. Therefore, we are confident that the results obtained are not biased due to actual sample size or effective size.

As expected, the marginal contributions of the local pig subpopulations assessed to either gene diversity or allelic richness were low (Supplementary Tables 2, 3, and 4). This can be explained by 3 reasons: a) the use of a large and admixed, including different genetic backgrounds (Fig. 2), Cosmopolitan pig population as a reference; b) the use of medium-density biallelic arrays data; and c) the little differentiation within geographical subsets (Figs. 1 and 2). Furthermore, the assessed subpopulations do not gather private alleles to a noticeable extent. The program Metapop2 gave estimates of private allele richness for each of the subpopulations assessed (not shown), reaching null (in most cases) or very low values (ranging from 0 to 0.00003). Note that, for the whole dataset, the higher contribution to gene diversity assessed was roughly 0.1%, and that for allelic richness about 0.3%. However, contributions to diversity assessed in the Asian and non-Asian pig subpopulations differ substantially (Figs. 3 and 4). This could be partially explained by the well-known separation between European (and American) and Chinese pig subpopulations (Megens et al., 2008). However, other features may contribute to such differences.

### The American-European scenario

Although European and American pig subpopulations are expected to be representative of a common genetic background, some structuring could be assessed via PCA (Fig. 1) and Admixture analyses (Fig. 2) within this subset. This structure is fully consistent with previous reports suggesting there exist 2 different genetic backgrounds in European pig breeds forming the so-called Mediterranean and Northerner clades (Megens et al., 2008; Burgos-Paz et al., 2013) with the Mangalitsa subpopulation clustering into the Mediterranean clade (Megens et al., 2008; Poklukar et al., 2023). This structuring can be partially explained considering the evidence suggesting that pig subpopulations of the Iberian Peninsula and Italy derive from local domestication events involving different wild boar populations from those used for the formation of Northern-Central European pig subpopulations (Larson et al., 2005). However, it has also been suggested that Iberian-type pigs were historically widespread in Europe, affecting Italian and some Eastern-Central European pig subpopulations such as Mangalitsa (Megens et al., 2008; Poklukar et al., 2023).

The analysis of the America-European subset of pig subpopulations only dramatically affected the contributions to the diversity assessed. Although still contributing favorably to gene diversity, a number of highly endangered pig subpopulations deriving from a small number of founders, with small population census and subject to relatively recent conservation programs (Brisbin and Sturek, 2009; Veroneze et al., 2014; Menéndez et al., 2016a; Kierkegaard et al., 2020) contributed unfavorably to allelic richness. A well-studied example is the Spanish Gochu Asturcelta pig breed, deriving from 4 founders only (Menéndez et al., 2016a; Arias et al., 2022). The fact that these breeds contribute favorably to gene diversity may be surprising. However, the formation of a population using founders that may have experienced local bottlenecks would lead to an excess of gene diversity in the subsequent generations (Álvarez et al., 2008) and can make difficult the assessment of autozygosity (Arias et al. 2023).

Another noticeable effect of using the America-European subset was the rearrangement of the pig subpopulations, contributing favorably to allelic richness only. When the whole dataset is used, contributions to gene diversity separate well the Mediterranean-like pig subpopulations, including Iberian, 2 Creole pig subpopulations (Peruvian and Yucatán minipig), which have been reported to gather the higher proportions of Iberian pig genetic background among the national American Creole pig subpopulations (Burgos-Paz et al., 2013), and 2 Italian subpopulations (Cinta Senese and Nera Siciliana) repeatedly reported as genetically close to Iberian pig (Megens et al., 2008; Yang et al., 2017; Poklukar et al., 2023). These subpopulations were separated from a) most other American-European pig subpopulations; b) Mediterranean pig subpopulations of expected origin in Northern-Central European pigs (Menéndez et al., 2016a; Arias et al. 2024); and c) Italian pig subpopulations such as the Calabrese and the Casertana pigs that have been reported to be genetically closer to Northern European pig subpopulations than to other Italian pig subpopulations, such as Cinta Senese, or Iberian pig (Poklukar et al., 2023). The results obtained using the whole dataset are appealing because they would fit well with the effect of possible local domestication events that occurred in both the Iberian and the Italian Peninsulas (Larson et al., 2005). However, it cannot be discarded that the results obtained using the whole dataset are biased due to the influence of the Asian pig subpopulations. When the American-European subset is used, the number of pig subpopulations contributing unfavorably to the 2 parameters assessed increased, adding pig subpopulations with small population sizes such as Creole Yucatán minipig, Spanish Manchado de Jabugo or American Guinea Hog to the set formed by the Italian Mora Romagnola, Hungarian Mangalitsa, and British Tamworth. However, the most noticeable reassessment affected several Italian pig subpopulations such as Mora Romagnola, Cinta Senese, Casertana, or Calabrese (Fig. 4A). Some of these results are difficult to explain: the Mangalitsa pig is expected to have been formed in the early 1800s by crossbreeding between Mediterranean pigs with Hungarian local pigs (Egerszegi et al., 2003; Marincs et al., 2013; Poklukar et al., 2023); and the Mora Romagnola pig has a history of crossbreeding with Duroc pig (Poklukar et al., 2023). Italian pig populations suffered a period of sharp decline during the first half of the twentieth century, and only in the 1990s did the recovery of these populations start in a structured and continuous way (Franci et al., 2005). In this phase of partial oblivion, interbreeding events may have occurred but mainly with cosmopolitan breeds (Franci and Pugliese, 2007). These introgression phenomena do not diminish the contribution of these breeds when considering the scenario that includes Asian populations. Still, analyzing only the American and European genotypes we observe a negative contribution to both genetic diversity and allelic richness.

### The Asian scenario

In the first view, the results obtained for the Asian pig subpopulations could be explained by the differences between Asian and American-European pigs. Differences exist in the extent of linkage disequilibrium, which is higher in Europe (Amaral et al., 2008). Moreover, using microsatellites, Chinese pig subpopulations were reported to have higher gene diversity values than European pig subpopulations (Yang et al., 2003; Megens et al., 2008).

However, this possible higher molecular diversity has not been confirmed using SNP array data, with Chinese pig subpopulations tending to have lower expected heterozygosity and allelic richness values than Cosmopolitan European pig subpopulations (Ai et al., 2013; Herrero-Medrano et al., 2014). Whether they are computed using either the whole dataset or the Asian subset, the contributions to diversity were highly consistent: no favorable contributions to allelic richness were assessed for the Chinese, Thai, or Korean local pig subpopulations (Figs. 3 and 4B). Although using the Asian subset allowed to identify several pig subpopulations contributing favorably to gene diversity, the pattern for allelic richness remained the same. Notably, both the Chinese and the non-Chinese Asian subpopulations had a lower number of alleles per locus than the European and American pig subpopulation groups (Table 1). Therefore, although this is hard to assume, it should be concluded that Asian pigs would not add favorably to the allelic richness of the domestic pig metapopulation worldwide.

The current results suggest that SNP calling quality may be lower in Asian samples than in American or European samples. Although most SNPs included in the PorcineSNP60 Beadchip amplify in Asian pig samples, this SNP array has been designed and validated using non-Asian pig samples (Ramos et al., 2009). Therefore, contributions to the diversity of Asian pig subpopulations can be severely biased and should be considered cautiously.

Moreover, although it has been reported that Northern Chinese pig subpopulations may differ in diversity from Central-Southern China pig populations (Yang et al., 2003; Megens et al., 2008), our results suggest that, if these differences exist, they are not reflected in the contributions of the Asian pig subpopulations to the diversity of the pig metapopulation.

### General discussion

Although uniqueness cannot be straightforwardly assessed from molecular data, contributions to diversity are usually computed for conservation purposes to control losses of genetic diversity while preserving allele frequencies of a given metapopulation (Zhao et al., 2021; Wang et al., 2022). However, the current work aims to assess how and to what extent local domestic pig subpopulations can be considered genetic reservoirs of domestic pig metapopulations worldwide. Although the relationship between functional diversity and overall diversity assessed using molecular markers is not clear (Groeneveld et al., 2010), the identification of putative reservoirs of the genetic diversity of the species may be important to accommodate future market and consumer demands in a domestic pig scenario overwhelmed by improved Cosmopolitan pig subpopulations.

Neither gene diversity nor allelic richness can accurately depict a worldwide domestic pig scenario. Instead, both parameters provide complementary information on the importance of an assessed subpopulation. Gene diversity is a requisite for selection and is commonly used in livestock conservation and subpopulation due to its direct correlation with genealogical coancestry (Gómez-Romano et al., 2016). However, preserving heterozygosity might not be enough to adequately preserve allelic richness, which is crucial for species persistence and evolution (Greenbaum et al., 2014). Maximizing gene diversity would cause marker alleles to keep intermediate frequencies, reducing the risk of loss due to genetic drift. However, this approach also puts rare alleles at risk of eventual loss (Fernández et al., 2004). Allelic richness provides information on the impact of selection, introgression, and genetic isolation on the genetic variability of a population (Rodrigáñez et al., 2008), as well as the long-term response to selection for adaptation to a changing environment and persistence (Caballero and García-Dorado, 2013; Greenbaum et al., 2014). Within the American-European subset, a few pig subpopulations well-known for being source of either high-quality food products or their ability to adapt to various environmental (altitude, hot climate) or extensive production conditions, such as the Iberian pig or the American Creole subpopulations (Silió, 2000; Burgos-Paz et al., 2013; Poklukar et al., 2023) have been characterized as favorable contributors to allelic richness no matter they contribute favorably to gene diversity or not. Therefore, allelic richness’s importance in identifying pig subpopulations as possible targets for conservation and characterization in genomic and production terms cannot be neglected.

Although differences in allelic richness can partially be explained by a different genetic origin of the subpopulations studied (Rodrigáñez et al., 2008), the largely divergent origin of the Asian pig is not reflected in favorable contributions to allelic richness. Stochastic processes can highly reduce allelic richness with no major effect on gene diversity due to the low influence of rare alleles on expected heterozygosity (Jordana et al., 2017). However, it is hard to assume that such stochastic events, occurring by definition at random, can affect all subpopulations in a country (China) and even a continent. One can argue that extensive gene flow between Asian and European ancestral subpopulations and between Cosmopolitan and local pig subpopulations worldwide has obscured the origin of modern subpopulations and the relationships between them (Chen et al., 2017), therefore affecting the assessment of allelic richness. However, the possibility of a biased performance of porcine SNP arrays when applied to Asian pig samples may be more parsimonious and cannot be rejected. The use of other, less biased, genotypic information, such as that provided by RAD Capture (Ali et al., 2016), can be advised to obtain a more realistic picture of the contributions of non-European pig populations to the diversity of the species.

## Conclusions

The current research suggests that local pig populations worldwide, as a whole, gather a non-negligible part of the genetic variability of the domestic pig metapopulation assessed both in terms of gene diversity (expected heterozygosity) and allelic richness. The contributions to allelic richness tend to be more consistent than those assessed for gene diversity, suggesting that the former may be a key parameter for the identification of candidate pig subpopulations for further conservation or characterization as putative carriers of rare genetic variants. However, results vary substantially among continental groups of subpopulations: Asian pig subpopulations did not contribute favorably to allelic richness. The possibility of a worse calling performance of the pig SNP arrays when used on Asian pig samples cannot be discarded and, therefore, the use of genotypic information obtained with other potentially less biased sources than SNP arrays can be advised when the aim is to compare the genetic diversity gathered by Asian and American-European pig subpopulations.

Furthermore, the results presented can be of interest to Genebank managers or researchers involved in the conservation of local animal genetic resources. It has been suggested that Genebanks should be made more dynamic by refining the criteria used for incorporating different biological materials (Paiva et al., 2016). Although production, geographic, and environmental criteria have been assayed (McManus et al., 2021), the use of marginal contributions to diversity has not been explored enough. Here, we propose to assess contributions to diversity in a livestock genetic scenario in which highly selected cosmopolitan populations overwhelm the genetic diversity of the local populations that are putatively in danger. Since the pool of Cosmopolitan pig breeds used as a reference in our study has the highest contribution values for both gene diversity and allelic richness, one can argue that there is no need to conserve local genetic stocks. On the contrary, the fact that small local populations still contribute favorably to the diversity gathered by a large-admixed population gives a clear idea of the putative importance of small local populations as reservoirs for the diversity of a given livestock species and to accommodate consumer demands in a changing scenario. Although marginal contributions to diversity may be small, local livestock populations contributing favorably to gene diversity and allelic richness should be prioritized to be included in Genebanks.

## Supplementary Material

skae329_suppl_Supplementary_Material

## Abbreviations

HS: within-subpopulation contribution to gene diversity
DG: between-subpopulations contribution to gene diversity
HT: total contribution to gene diversity
AS: within-subpopulation contribution to allelic richness
DA: between-subpopulations contribution to allelic richness
AT: total contribution to allelic richness
Ne: effective subpopulation size
Ne(M): effective subpopulation size estimated molecular coancestry
Ne(LD): effective subpopulation size estimated linkage disequilibrium information
